# BS69/ZMYND11 C-Terminal Domains Bind and Inhibit EBNA2

**DOI:** 10.1371/journal.ppat.1005414

**Published:** 2016-02-04

**Authors:** Matthew R. Harter, Cheng-Der Liu, Chih-Lung Shen, Elsie Gonzalez-Hurtado, Zhi-Min Zhang, Muyu Xu, Ernest Martinez, Chih-Wen Peng, Jikui Song

**Affiliations:** 1 Department of Biochemistry, University of California, Riverside, Riverside, California, United States of America; 2 Institute of Medical Sciences, Tzu Chi University, Hualien, Taiwan; 3 MARC U-STAR Program, University of California, Riverside, Riverside, California, United States of America; Wistar Institute, UNITED STATES

## Abstract

Epstein-Barr virus (EBV) nuclear antigen 2 (EBNA2) plays an important role in driving immortalization of EBV-infected B cells through regulating the expression of many viral and cellular genes. We report a structural study of the tumor suppressor BS69/ZMYND11 C-terminal region, comprised of tandem coiled-coil-MYND domains (BS69_CC-MYND_), in complex with an EBNA2 peptide containing a PXLXP motif. The coiled-coil domain of BS69 self-associates to bring two separate MYND domains in close proximity, thereby enhancing the BS69 MYND-EBNA2 interaction. ITC analysis of BS69_CC-MYND_ with a C-terminal fragment of EBNA2 further suggests that the BS69_CC-MYND_ homodimer synergistically binds to the two EBNA2 PXLXP motifs that are respectively located in the conserved regions CR7 and CR8. Furthermore, we showed that EBNA2 interacts with BS69 and down-regulates its expression at both mRNA and protein levels in EBV-infected B cells. Ectopic BS69_CC-MYND_ is recruited to viral target promoters through interactions with EBNA2, inhibits EBNA2-mediated transcription activation, and impairs proliferation of lymphoblastoid cell lines (LCLs). Substitution of critical residues in the MYND domain impairs the BS69-EBNA2 interaction and abolishes the BS69 inhibition of the EBNA2-mediated transactivation and LCL proliferation. This study identifies the BS69 C-terminal domains as an inhibitor of EBNA2, which may have important implications in development of novel therapeutic strategies against EBV infection.

## Author Summary

Since the discovery of Epstein-Barr virus (EBV) 50 years ago, the etiologic links between EBV and a variety of human cancers have gained wide recognition. It is estimated that >90% of the worldwide population carry this virus, which causes over 200,000 cancers across the world every year. One of the key proteins in driving immortalization of EBV-infected B cells is Epstein-Barr virus nuclear antigen 2 (EBNA2), which regulates the expression of many cellular and viral genes. However, the molecular mechanism underlying the interactions between EBNA2 and cellular transcriptional regulators remains enigmatic. Here, we determined the crystal structure of the coiled-coil and MYND tandem domains of BS69/ZMYND11, a candidate tumor suppressor, in complex with an EBNA2 peptide containing a PXLXP motif. We found that the coiled-coil and MYND domains of BS69 cooperate in binding to EBNA2. We also showed that EBNA2 interacts with BS69 and down-regulates its expression at both mRNA and protein levels in EBV-associated B cells. Ectopic BS69 coiled-coil-MYND dual domain is recruited to viral target promoters through interaction with EBNA2, inhibits EBNA2-mediated transcription activation, and impairs proliferation of lymphoblastoid cell lines (LCLs). Together, this study identifies the BS69 C-terminal domains as an inhibitor of EBNA2.

## Introduction

**Fig 1:**
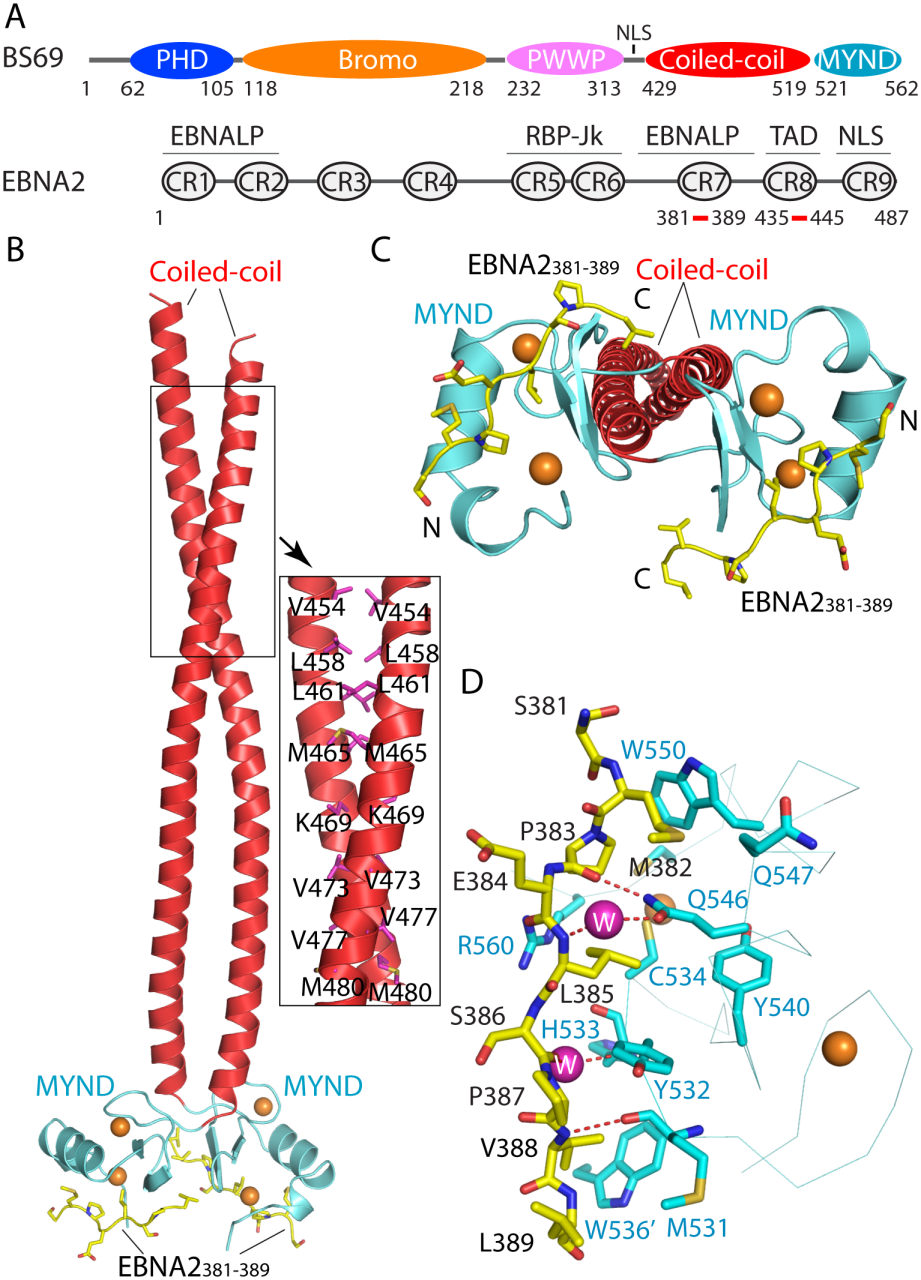
Structural analysis of the BS69_CC-MYND_ domains in complex with the EBNA2_381–389_ peptide. (A) Domain architecture of BS69 and EBNA2. Regions responsible for EBNA-LP activation or RBP-Jk binding, transactivation domain (TAD) and nuclear localization sequence (NLS) of EBNA2 are labeled. Note that CR5 contributes to RBP-Jk binding only indirectly [16]. (B) Ribbon representation of the BS69_CC-MYND_ domains (coiled-coil: red; MYND: light blue) in complex with the EBNA2_381–389_ peptide (yellow sticks). The zinc ions are shown in orange spheres. Selected intermolecular interactions from the coiled-coil domain are shown in the expanded view. (C) Structure of the BS69_CC-MYND_-EBNA2_381–389_ complex in a view different from (B). (D) Close-up view of the BS69_CC-MYND_-EBNA2_381–389_ interactions. The hydrogen bonds are shown in dashed line. The water molecules are shown in magenta spheres. Residue W536 from the neighboring MYND domain is labeled with an apostrophe mark.

Epstein-Barr virus (EBV) is a widespread herpes virus that transforms resting B cells into permanent lymphoblastoid cell lines [1, 2]. Under some circumstances, this may further lead to several malignancies, including Burkitt’s lymphoma, Hodgkin lymphoma and nasopharyngeal carcinoma [3]. One of the key EBV proteins that drive immortalization of B cells is Epstein-Barr virus nuclear antigen 2 (EBNA2). It is, together with another EBV protein, EBNA-LP, the first protein to be expressed upon infection [4, 5]. These two proteins then cooperate to promote the G0–G1 phase transition of the cell cycle [6]. EBNA2 plays a critical role in controlling the expression of many viral and cellular genes [7]. For instance, it recruits a variety of cellular proteins, including histone acetyltransferases (e.g. P300) [8] and basal transcription factors [9–11], to regulate chromatin structure and gene expression. Sequence comparison of EBNA2 across serotypes of EBV, combined with mutational studies, has identified nine evolutionarily conserved regions (CR1-CR9) (Fig 1A) that define the functional domains of EBNA2 [12]. Most notably, CR8 (residues 437–477) is the transactivation domain (TAD) [12], which interacts with both acetyltransferases and EBNA-LP to mediate transcriptional activation [8, 13, 14]; CR5 and CR6 mediate indirect contact of EBNA2 with DNA [15, 16]. In addition, several other domains, including CR7, are important for EBNA2-LP coactivation [17, 18].

BS69 (ZMYND11) is an emerging tumor suppressor [19–21] that was originally identified as the Adenovirus protein E1A and EBNA2-interacting protein [22, 23]. It has been shown that low expression of BS69 correlates with poor prognosis in breast cancer patients, whereas its overexpression suppresses cancer cell growth both *in vitro* and *in vivo* [21]. BS69 contains, in addition to a C-terminal MYND (MYeloid translocation protein 8, Nervy and DEAF-1) domain, an N-terminal Plant Homo Domain (PHD) zinc finger, a Bromo domain and a PWWP domain. Recent studies have demonstrated that the tandem Bromo-PWWP domains of BS69 specifically recognize histone H3.3 trimethylated at lysine 36 (H3.3K36me3), thereby linking BS69 to transcriptional elongation, tumor suppression and pre-mRNA splicing [19, 21]. The structure and function of the BS69 C-terminal region is less clear. Nevertheless, it has been reported that BS69 interacts with E1A, EBNA2 and a variety of cellular transcriptional regulators through its MYND domain [20, 22–25], which recognizes a common PXLXP (X denotes any amino acid) sequence motif present in many of these proteins [22].

In this study, we identified a coiled-coil domain that precedes the MYND domain of BS69. The crystal structure of the coiled-coil domain in tandem with the MYND domain of BS69, bound to an EBNA2 peptide (residues 381–389, EBNA2_381–389_), reveals that BS69 forms a homodimer through the self-association of its coiled-coil domain, permitting the two MYND domains of the BS69 dimer to cooperatively bind to the EBNA2_381–389_ peptide. Through ITC assays, we also demonstrated that the BS69 homodimer binds to the two PXLXP motifs within CR7 and CR8 of EBNA2 in a synergistic manner. Interestingly, analysis of the EBV-infected B cells indicates that BS69 interacts with EBNA2 at the early stage of EBV infection, but is subsequently suppressed by EBNA2 at both mRNA and protein levels. We confirmed that the ectopically expressed BS69 coiled-coil-MYND domains can interact with EBNA2 in cells through a co-immunoprecipitation (co-IP) assay. Furthermore, through *in vivo* transcription reporter and cell viability assays, we showed that ectopic expression of the BS69 coiled-coil-MYND domains leads to inhibition of the EBNA2-mediated transcriptional activation in the EBNA2-transfected BJAB B lymphoma cell line [26], and decreased viability of lymphoblastoid cell lines (LCLs). By contrast, substitution of critical residues in the MYND domain disrupts the BS69-EBNA2 interaction and abolishes the BS69 inhibition of both the EBNA2-mediated transactivation and LCL cell growth. Taken together, this study identifies the BS69 C-terminal domains as a potential inhibitor of EBNA2, thereby providing a mechanism for development of novel therapeutic strategies against EBV infection.

## Results

### Structural characterization of the BS69-EBNA2 interaction

A previous study suggested that the interaction between BS69 and EBNA2 is mediated by the MYND domain of BS69 (residues 521–562) and the PXLXP motif from EBNA2 [22]. However, sequence analysis of BS69 using the program Paircoil2 [27] suggests that this protein may also contain a coiled-coil domain (residues 429–520), immediately preceding its C-terminal MYND domain (Fig 1A). Therefore, we have set out to investigate the interaction of a C-terminal fragment of BS69, comprised of both the predicted coiled-coil domain and the MYND domain (BS69_CC-MYND_), with a PXLXP motif-containing peptide derived from region CR7 of EBNA2 (residues 381–389, EBNA2_381–389_) (Fig 1A). The crystal structure of BS69_CC-MYND_ (residues 440–562) in complex with EBNA2_381–389_ was determined at 2.4 Å resolution (Fig 1B and 1C). The structure of the BS69_MYND_ domain reveals a ββα fold that was observed for other MYND domains [28, 29]. Two zinc finger clusters, formed by a Cys_4_ motif and a Cys_3_His motif, respectively, are arranged in a cross brace topology. In addition, a short 3_10_-helical turn (α2) immediately follows the major α-helix (α1), participating in formation of one of the zinc clusters. Structural analysis of the BS69_CC-MYND_-EBNA2_381–389_ complex also confirmed the presence of a coiled-coil domain upstream to the MYND domain (Fig 1B). The coiled-coil domains from two BS69_CC-MYND_ molecules further associate to form a homodimeric fold. Consequently, the two MYND domains within the same BS69_CC-MYND_ dimer are brought in close proximity (Fig 1B and 1C). Association of BS69_CC-MYND_ with EBNA2_381–389_ is mainly mediated through hydrogen bonds and van der Waals contacts, involving a surface area formed by the α1-helix and β1-strand of BS69_CC_-_MYND_ (Fig 1C and 1D). Notably, the side chain of BS69 Q546 forms direct and water-mediated hydrogen bonds with the backbone atoms of EBNA2 P383 and L385, respectively; and BS69 M531 and H533 form direct and water-mediated backbone hydrogen bonds with EBNA2 V388 and S386, respectively. The three conserved residues in the PXLXP motif of EBNA2_381–389_: P383, L385 and P387, all make contacts with BS69_CC-MYND_ (residues Y532, C534, Y540, Q546, W550, C558 and R560) through van der Waals contacts (Fig 1D). Additional intermolecular contacts involve the residues flanking the PXLXP motif: EBNA2 M382 makes contacts with BS69 Q546, Q547 and W550, and EBNA2 V388 is in close proximity with W536 from a second MYND domain within the same BS69 dimer (Fig 1D). The engagement of W536 from the neighboring MYND domain in the BS69_MYND_-EBNA2_381–389_ interaction implies that the BS69 coiled-coil domain plays a role in enhancing the interaction between the BS69_MYND_ domain and EBNA2.

### ITC analysis of the BS69-EBNA2 interaction

**Fig 2:**
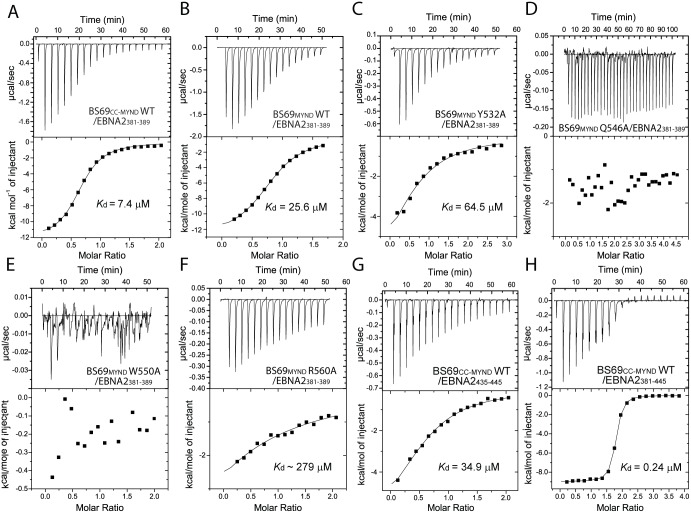
(A-H) ITC analysis of BS69 wild type and mutants binding to the EBNA2 peptides. In (A-G) EBNA2 peptides were titrated against BS69. In (H) BS69_CC-MYND_ was titrated against EBNA2_381–445_. For the R560A mutant, the *K*_d_ value was estimated by fixing the parameter of the stoichiometric ratio (N) to 1.

To test our structural observation, we performed mutational studies of BS69 and Isothermal Calorimetry Titration (ITC) assays to evaluate the BS69-EBNA2 binding (Fig 2 and S1 Fig). We observed that BS69_CC-MYND_ and BS69_MYND_ bind to the EBNA2_381–389_ peptide with a dissociation constant (*K*_d_) of 7.4 μM and 25.6 μM, respectively (Fig 2A and 2B and S1A Fig), confirming that the coiled-coil domain of BS69 contributes to its binding to EBNA2. In addition, we showed that mutation of Y532 and R560 of BS69_MYND_ each to alanine decreased the BS69_MYND_-EBNA2_381–389_ binding by about 3- and 10-fold, respectively (Fig 2C and 2F and S1A Fig). Even more dramatically, mutation of Q546 and W550 of BS69_MYND_ largely abolished the interaction between BS69_MYND_ and EBNA2_381–389_ (Fig 2D and 2E). Together, these data lend a strong support for our structural observation of the BS69_CC-MYND_-EBNA2_381–389_ complex.

A previous study based on GST pull-down assay suggests that the BS69_MYND_ domain may also interact with another PXLXP motif from region CR8 of EBNA2 [22]. To gain further insight, we characterized the interactions between BS69 and an EBNA2 peptide encompassing this PXLXP motif (residues 435–445, EBNA2_435–445_). Our results showed that BS69_CC-MYND_ and BS69_MYND_ both bind to the EBNA2_435–445_ peptide, with a *K*_d_ of 34.9 μM and 93.4 μM, respectively (Fig 2G and S1B Fig). To determine whether the two PXLXP motifs in EBNA2 cooperate in BS69 association, we also measured the binding affinity of BS69_CC-MYND_ for an EBNA2 fragment (residues 381–445, EBNA2_381–445_) encompassing both PXLXP motifs. As shown in Fig 2H, BS69_CC-MYND_ binds to EBNA2_381–445_ with a *K*_d_ of 0.24 μM and a monophasic binding curve, which is 30–150 fold stronger than the binding of BS69_CC-MYND_ to either motif alone. Therefore, these data suggest that the two monomers of the BS69_CC-MYND_ homodimer synergistically bind to the two sequential PXLXP motifs of EBNA2, resulting in enhanced BS69-EBNA2 recognition (S2 Fig). On the other hand, the Q546A mutation substantially decreases the respective binding affinities of the BS69_MYND_-EBNA2_435–445_ and BS69_CC_-_MYND_-EBNA2_381–445_ complexes (S1C and S1D Fig), similar to what was observed for the BS69_MYND_-EBNA2_381–389_ interaction. Furthermore, a Q546A/W550A double mutation largely abolishes the binding between BS69_CC-MYND_ and the EBNA2_381–445_ peptide (S1E Fig).

### Comparison of the protein interactions mediated by BS69_MYND_ and other MYND domains

**Fig 3:**
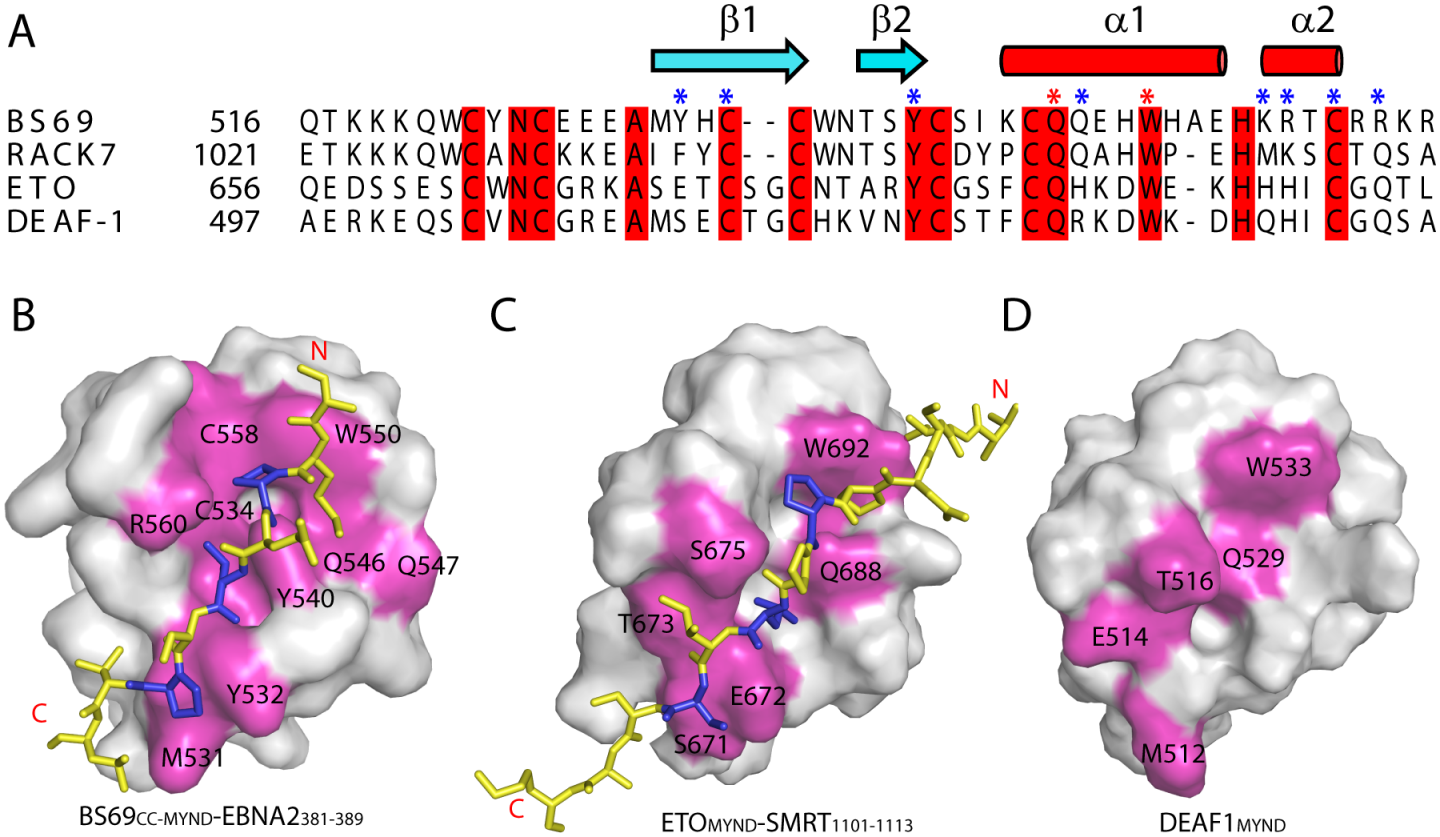
Comparison of the BS69_MYND_-EBNA2_381–389_ interaction with those of other MYND domains. (A) Structure-based sequence alignment of BS69_MYND_ with the MYND domains from other proteins. Residues Q546 and W550 of BS69_MYND_, marked with red asterisk, were mutated for *in vivo* functional assay. The other EBNA2-interacting residues of BS69_MYND_ are marked with blue asterisks. (B-D) Surface view of the BS69_MYND_ with the EBNA2_381–389_ peptide (B), the ETO_MYND_ domain in contact with SMRT_1101–1113_ (C), and the DEAF1_MYND_ domain (D), with the protein interaction sites highlighted in magenta. EBNA2_381–389_ P383, L385 and P387, and the corresponding residues in SMRT_1101–1113_ are highlighted in blue in (B) and (C), respectively. Note that the BS69_MYND_-EBNA2_381–389_ interaction is enhanced by dimerization of the BS69 coiled-coil domain.

In addition to BS69, the MYND domain exists in a variety of chromatin-related proteins [30]. Currently, structural information on the interactions between this class of domains and their binding partners has only been limited to leukaemogenic protein AML1-ETO [29] and transcriptional regulator DEAF-1 [28]. An NMR structural study of the AML1-ETO MYND domain (ETO_MYND_) revealed that it recognizes a PPPLI sequence motif, present in nuclear co-repressors SMRT and N-CoR, through antiparallel β-pairing interactions [29]. Using NMR titrations, a similar binding model was later revealed for the interactions of the DEAF-1 MYND domain with SMRT and N-CoR [28]. Our structure-based sequence alignment of BS69_MYND_ with these reported MYND domains, as well as its closely related RACK7 MYND domain (RACK7_MYND_), reveals that the overall sequence identity between these MYND domains is only around 30%, the majority of which are the cysteine and histidine residues that coordinate the zinc ions (Fig 3A). Nevertheless, the protein interaction sites of BS69_MYND_, AML1-ETO MYND domain (ETO_MYND_) and DEAF-1 MYND domain (DEAF-1_MYND_) appear to be highly conserved, with each formed by the α1-helix and β1-strand of the respective proteins (Fig 3B–3D). Of particular note, three strictly conserved, non-zinc binding residues (Y540, Q546 and W550 in BS69_MYND_) are important for mediating the protein interactions of all three proteins [28, 29]. For instance, the BS69_MYND_ Q546 and W550 equivalent residues in ETO_MYND_ (Q688 and W692, respectively) interact with SMRT_1101–1113_ through hydrogen bond formation and packing against a proline residue from SMRT_1101–1113_, respectively (Fig 3C) [29], in a similar fashion to what is observed for BS69 Q546 and W550 in the BS69_CC_-_MYND_-EBNA2_381–389_ complex (Fig 1D). The equivalent residues in DEAF-1_MYND_ (Q529 and W533, respectively) also underwent large chemical shift perturbations in DEAF-1_MYND_ when peptides from nuclear co-repressor N-CoR or SMRT are present (Fig 3D) [28]. Together, these observations demonstrate that MYND domains, despite having large sequence variation, adopt evolutionarily conserved surface sites for protein interaction. On the other hand, we also observed a number of BS69_MYND_-unique protein interaction sites (e.g. Y532, Q547, R560) (Fig 3A and 3B), which help define the surface complementarity between BS69_MYND_ and EBNA2_381–389_, and provide the molecular basis for the binding specificity of the BS69_MYND_ domain, as suggested by a previous molecular modeling analysis [28].

### Expression analysis of BS69 in EBV-infected B cells

**Fig 4:**
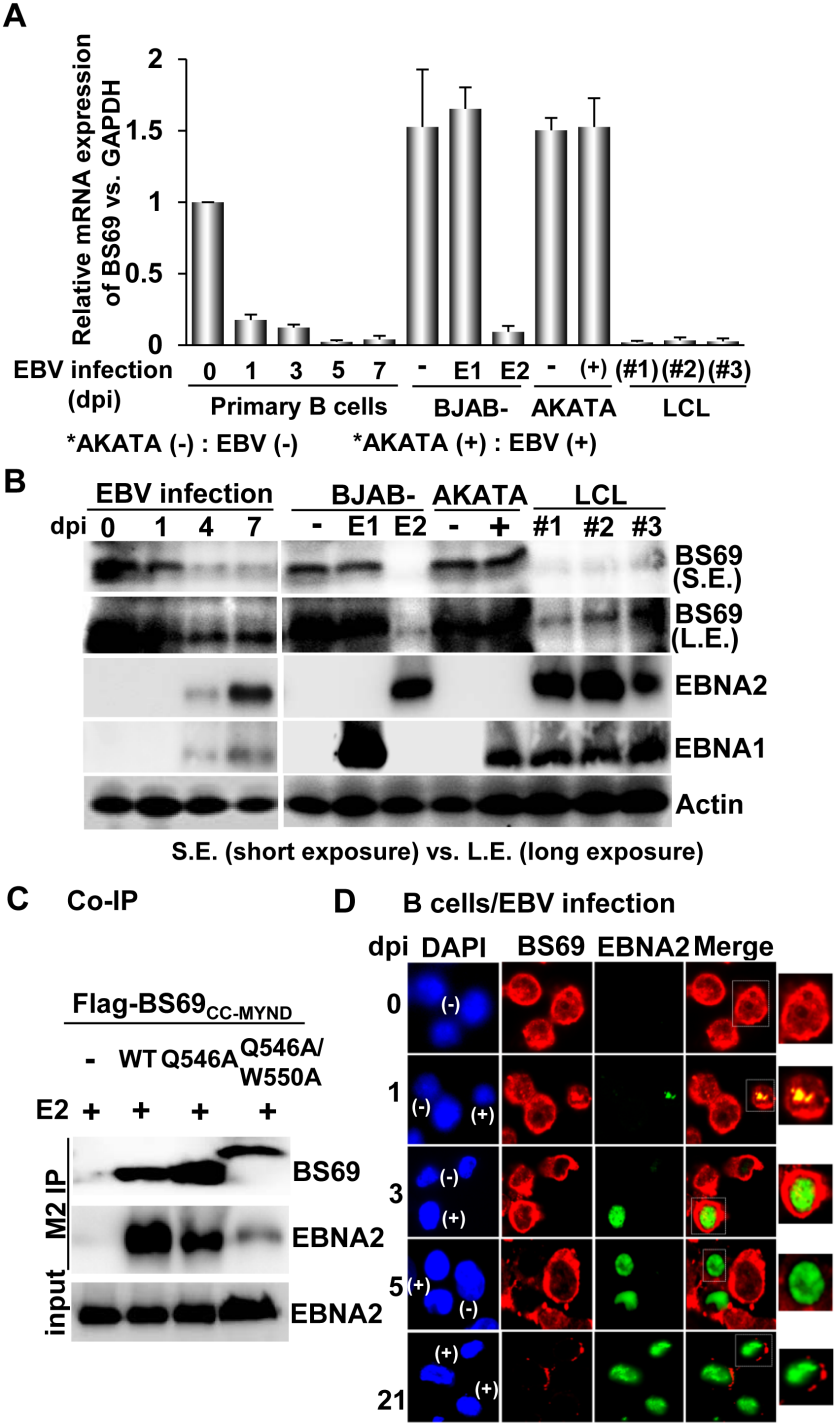
Expression and interaction analysis of BS69 and EBNA2 in EBV-infected cells. (A) The relative mRNA expression of endogenous BS69 over GAPDH in different EBV-infected cell lines or B cells with EBV-infection at various days of post-infection (dpi) was identified by qRT-PCR. (B) Expression of endogenous BS69, EBNA2 and EBNA1 in B cells with EBV infection at 0, 1, 4, and 7 dpi, LCLs, and B lymphoma cells was analyzed by Western blotting using antibodies against BS69, EBNA2 and EBNA1, respectively. The images with short exposure (S.E.) versus long exposure (L.E.) were shown. The expression of β-actin was used as internal control. (L.E.) (C) Transfection-mediated Co-IP analysis was performed using BJAB cells co-transfected with the plasmids of flag-BS69_CC-MYND_ wild type, Q546A, Q546A/W550A and EBNA2 (E2). The anti-flag M2 antibody was used for immunoprecipitation of flag-BS69_CC-MYND_ wild type or Q546A. The slower migration of the Q546A/W550A band was due to the fact that its linker sequence bridging the flag-tag and BS69_CC-MYND_ is 13-amino acid longer than that of wild type or Q546A. (D) Resting primary B cells with the indicated dpi of EBV infection were used to evaluate the EBNA2 and BS69 expression and localization by confocal immunofluorescence microscopy. The nuclei were counterstained with DAPI (blue). Both individual and merge images were shown. EBNA2 was shown in green versus BS69 was shown in RED. Cells infected with EBV were indicated as (+) while uninfected cells were indicated as (-). The enlarged view of the marked regions were shown, respectively.

A previous study has indicated that EBNA2 is recruited to the BS69 promoter upon EBV infection of B cells [31]. Along this line, we ask whether EBNA2 influences the expression of BS69 in EBV-infected cells. For this, we performed quantitative real time PCR to monitor the changes of the endogenous BS69 mRNA levels in B cells with EBV infection from 0 to seven days of post-infection. Prominently, we observed that the relative mRNA level of BS69 over Glyceraldehyde 3-phosphate dehydrogenase (GAPDH) in primary B cells was significantly decreased following EBV infection, with a >20-fold reduction at day 5 post-infection (Fig 4A). Among a number of EBV-infected cell lines, a large down-regulation of the BS69 mRNA level was also observed for three independent immortalized LCLs (Fig 4A). On the other hand, the BS69 mRNA remained expressed abundantly in the EBV latency I AKATA cells [32], regardless of the presence of EBV genome (Fig 4A). Given the fact that EBNA2 is not expressed in EBV-infected AKATA cells [32], we postulate that the presence of EBNA2 leads to down-regulation of the BS69 expression in the EBV-infected primary B cells and LCLs. We therefore analyzed the BS69 mRNA from EBV-negative B lymphoma cell line, BJAB, and two of BJAB derivative stable clones, BJAB-E1 and BJAB-E2, with constitutive expression of Epstein-Barr virus Nuclear Antigen 1 (EBNA1) and EBNA2, respectively. As shown in Fig 4A, the BS69 mRNA is expressed in parental BJAB and BJAB-E1, but downregulated in BJAB-E2, indicating that its expression is abrogated by the presence of EBNA2. Consistently, Western blotting analysis of the BS69 protein in EBV-infected B cells, B lymphoma or lymphoblastoid cells revealed that the protein level of BS69 became barely detectable in those B cells with EBV infection at 4 or 7 dpi and the BJAB-E2 or LCL cells, but persisted in the primary B cells, parental BJAB, BJAB-E1 and AKATA cells (Fig 4B). Together, these data suggest that EBNA2 down-regulates the expression of BS69 in EBV-infected B cells.

### Co-immunoprecipitation (Co-IP) analysis of the BS69_CC-MYND_-EBNA2 interaction

To address whether the BS69 C-terminal domain, if expressed, interacts with EBNA2 *in vivo*, we ectopically co-expressed flag-tagged wild-type, Q546A, or Q546A/W550A BS69_CC-MYND_ with EBNA2 in BJAB cells, followed by immunoprecipitation using anti-flag M2-conjugated sepharose and Western blotting with antibodies against EBNA2 and M2, respectively. As shown in Fig 4C, EBNA2 co-precipitates with wild-type flag-tagged BS69_CC-MYND_, indicating that BS69_CC-MYND_ and EBNA2 indeed can form a complex in cells. By contrast, the Q546A and Q546A/W550A BS69_CC-MYND_ mutants exhibits a ~50% and ~85% reduction of EBNA2 binding, confirming our structural and ITC analysis that BS69 Q546 and W550 are important for the BS69-EBNA2 interaction. Of importance, we showed that the colocalization of EBNA2 and BS69 appeared in EBV infected B cells at 1 and 3 dpi (Fig 4D), implicating the interaction of two proteins could take place in the host cells. In addition, the immunofluorescence (IF) image pointed to a reduction of large amount of BS69 expression in EBV infected B cells at 5 dpi, confirming the barely low expression levels of BS69 shown in Fig 4B.

### BS69_CC-MYND_ inhibits EBNA2-mediated transcriptional activation

**Fig 5:**
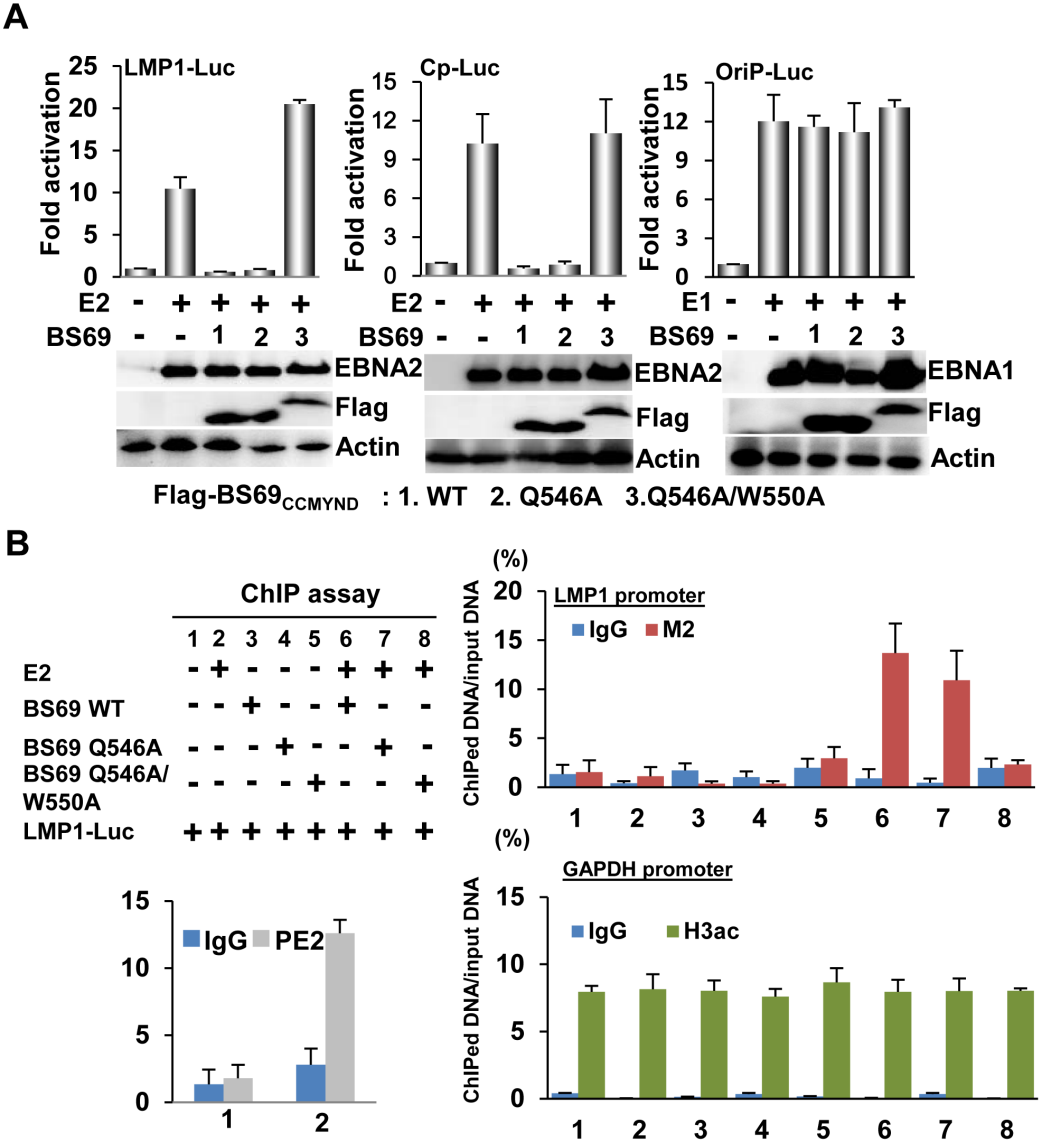
Recruitment of BS69_CC-MYND_ to EBNA2 target promoter through protein interactions leads to down regulation of EBNA2 dependent transcription. (A) EBNA2 specific reporter plasmids, LMP1-Luc and Cp-Luc, EBNA1 specific oriP-Luc reporter plasmid, CMV-βGal internal control, and the indicated expression plasmids were subjected to a procedure of transfection-mediated transcription reporter assay. The effects of flag-BS69_CC-MYND_ wild type, Q546A or Q546A/W550A on EBNA2-mediated transcription were determined by the resulting luciferase activity corrected for β-gal activity. (B) The experimental design of the transfection-mediated ChIP assay was shown. M2-conjugated sepharose was used to precipitate flag-BS69_CC-MYND_ wild type, Q546A, or Q546A/W550A while H3ac was used to precipitated acetylated-H3. PE2 (EBNA2) ChIP was used to assay EBNA2 enrichment at transfected LMP1 DNA. IgG was used as negative control. The amount of ChIPed DNA was quantified by real time PCR. The enrichment of BS69_CC-MYND_ wild type, Q546A, or Q546A/W550A at the LMP1 promoter and the enrichment of H3ac at GAPAH promoter were represented as % of input DNA, respectively.

Next, we ask whether the BS69 C-terminal domains, when expressed, influence the EBNA2-mediated transcriptional regulation. Toward this, we performed luciferase reporter assays on the EBNA2 target promoters, including LMP1 [33, 34] and BamHI C promoter (Cp) [35, 36]. In the absence of BS69, the EBNA2 protein activated a co-transfected LMP1-luciferase (LMP1-Luc) or Cp-luciferase (Cp-Luc) reporter genes both by around 10-fold (Fig 5A), confirming the strong transcriptional activation potential of EBNA2 [37]. The presence of wild-type BS69_CC-MYND_ reduced the relative transcriptional activation to 2-fold or less (Fig 5A), suggesting that BS69 down-regulates the EBNA2-mediated transcriptional activation. On the other hand, the presence of BS69 failed to suppress the EBNA1-mediated transcriptional activation of OriP (origin of replication)-Luc reporter, suggesting that BS69 specifically inhibits EBNA2. The BS69_CC-MYND_ Q546A mutation partially reduced its inhibition of the EBNA2-mediated transcriptional activation (Fig 5A), presumably due to the residual binding affinity of this mutant toward EBNA2. By contrast, the BS69_CC-MYND_ Q546A/W550A double mutation lost the inhibitory effect on EBNA2-mediated transcriptional activation. To further delineate the functional consequence of the BS69 Q546A mutation, we next transfected various amounts of wild-type or Q546A BS69_CC-MYND_ vector into HeLa cells and tested the effects on activation by a co-transfected C-terminal fragment of EBNA2 (residues 375–465, containing both CR7 and CR8) fused to the Gal4 DNA-binding domain (Gal4-EBNA2) using the G5-TK-luciferase (G5-TK-Luc) as a reporter gene [38]. Our choice of the EBNA2 (375–465) construct was based on a previous study [18] showing that this fragment exhibits a stronger transcriptional activation potential than the full-length protein. Indeed, we observed that the GAL4-EBNA2 fusion protein activated the G5-TK-Luc reporter gene by over 100-fold in the absence of BS69 (S3A and S3B Fig). However, in the presence of wild-type BS69_CC-MYND_ the transcriptional activation was reduced to less than 4-fold (S3B Fig), supporting the inhibitory role of BS69 in the EBNA2-mediated transcriptional activation. By contrast, a 60-fold activation by Gal4-EBNA2 was observed in the presence of the Q546A mutant (S3B Fig), and this mutant remained less repressive than wild type even at 5-fold excess (S3C Fig). To decipher the importance of the PXLXP motifs in BS69 inhibition of EBNA2 dependent transcription, we next introduced the EBNA2 PXLXP mutations, in which the proline residues within either of the two PXLXP motifs were replaced by alanine residues. Although mutations on either PXLXP motif did not affect the expression levels and transactivation function of EBNA2 (S4A Fig), mutations on the second PXLXP motif relieved ~50% of the BS69 inhibitory effect, while mutations on the first PXLXP motif had no effect (S3B–S3D Fig). These observations suggest that the BS69-EBNA2 interaction is required for BS69 inhibition of EBNA2-mediated transcriptional activation.

To determine whether BS69_CC-MYND_ is able to associate with EBNA2 target promoters through protein-protein interactions, we performed Chromatin Immunoprecipitation (ChIP) assays using BJAB cells, in which flag-tagged wild-type, Q546A, or Q546A/Q550A BS69_CC-MYND_ was co-transfected with or without EBNA2 expression vector and a reporter plasmid containing the LMP1 promoter, LMP1-Luc. In the absence of EBNA2, the amount of LMP1-DNA brought down by wild-type, Q546A or Q546A/Q550A BS69_CC-MYND_ ChIP is similar to that of IgG negative control (7–8% over input DNA) (Fig 5B), indicating no appreciable BS69_CC-MYND_-LMP1 DNA binding. On the other hand, an EBNA2 ChIP brought down ~13% of LMP1-DNA, consistent with the fact that LMP1 is one of the target sites of EBNA2 [35, 36]. Notably, the presence of EBNA2 also increases the amount of wild-type and Q546A BS69_CC-MYND_ ChIPed LMP1-DNA to ~17% and ~12%, respectively (Fig 5B), indicating that BS69_CC-MYND_ binding to LMP1-DNA is EBNA2 dependent. By contrast, the BS69 Q546A/Q550A mutation reduces the LMP1-DNA brought down by BS69_CC-MYND_ to a level similar to that of IgG negative control (Fig 5B). These data suggest that EBNA2 can recruit BS69_CC-MYND_ to its target sites through direct protein interactions, which therefore establishes BS69 C-terminal domains as a potential inhibitor of EBNA2.

### BS69_CC-MYND_ blocks EBNA2-mediated LCL proliferation

**Fig 6:**
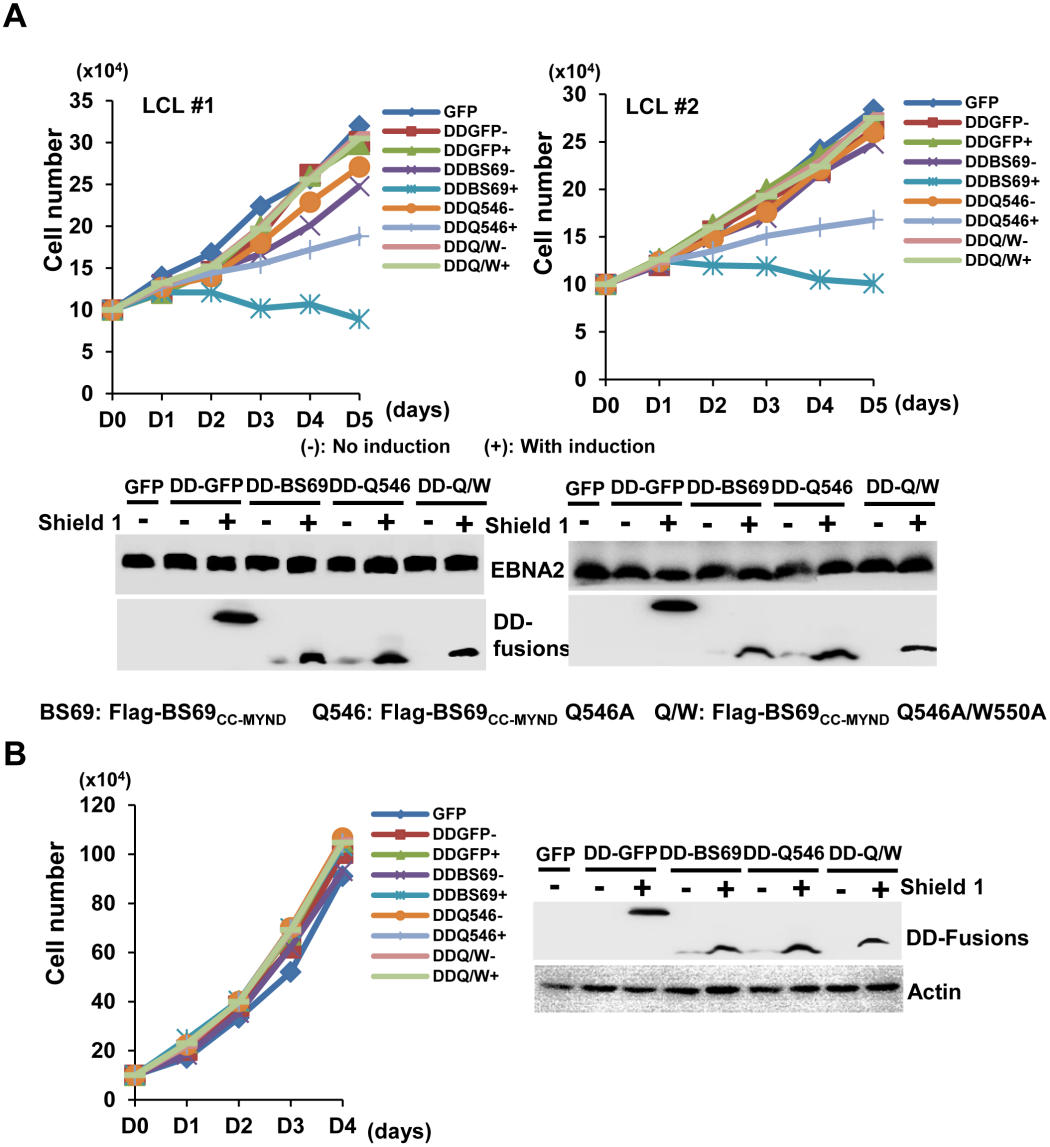
Induced expression of BS69_CC-MYND_ in LCLs leads to a strong debilitation of cell growth. (A) The retroviral vector mediated inducible expression vector of DD-BS69_CC-MYND_ wild type, Q546A, or Q546A/W550A was used to transduce two LCLs, LCL#1 and LCL#2, and BJAB control cell lines. DD-BS69: DD-BS69 wild-type; DD-Q546: DD-BS69 Q546A; DD-BS69 Q/W: DD-BS69 Q546A/W550A (B) 10^5^ per mL of the above retrovirus transduced cell lines were aliquoted into 6-well plates and cell viability assays were conducted with the treatment of inducer (shield 1) or PBS (negative control) every 24 hrs for five consecutive days. The expression of actin was used internal control.

It has been established that EBNA2 is essential for EBV-mediated B cell immortalization [39]. To determine whether introduction of the BS69-EBNA2 interaction could affect the proliferative activity of LCL, we generated an inducible retroviral vector harboring a destabilization domain (DD) N-terminally fused to wild type or Q546A flag-tagged BS69_CC-MYND_. The expression of the DD-BS69_CC-MYND_ fusion protein, wild-type or Q536A, can therefore be regulated by the ligand Shield 1, which binds to DD to keep the fusion protein from degradation [40]. Cell viability assays were performed on two independent LCLs transduced with the retrovirus vector encoding DD-BS69, wild-type, Q546A or Q546A/W550A, DD-GFP fusion protein or GFP alone (Fig 6A). We observed that the Shield 1-induction led to a complete loss in viability of both LCLs transduced with the DD-BS69_CC-MYND_ wild-type vector. The Q546A single mutation partially impaired the cell viability. By contrast, no appreciable change in viability occurred for the LCLs transduced with the DD-Q546A/W550A, DD-GFP or GFP vectors. On the other hand, the viability of the EBV-negative BJAB cells transduced with any of these vectors was not affected by Shield 1 induction, suggesting that the BS69 inhibition of cell viability is EBNA2-dependent (Fig 6B), either through direct inhibition of EBNA2 function or interference with its downstream signaling events (e.g. LMP1 signaling). Together, these data suggest that introduction of the interaction between BS69_CC-MYND_ and EBNA2 can lead to inhibition of the EBNA2-mediated cell proliferation.

## Discussion

In this study, we determined the crystal structure of the BS69_CC-MYND_-EBNA2_381–389_ complex, which provides important insights into how BS69 recognizes the common PXLXP motif, present in EBNA2, E1A and a variety of cellular transcriptional regulators. Importantly, dimerization of the BS69 coiled-coil domain results in an enhanced and synergistic interaction between BS69 and the two sequentially proximate EBNA2 PXLXP motifs, located in CR7 and CR8, respectively. Note that both CR7 and CR8 of EBNA2 play a role in its oncogenic function: CR7 is important for coactivation of EBNA2 by EBNALP protein [17, 18], while CR8, the transcriptional activation domain of EBNA2, mediates gene transcriptional activation through its recruitment of cellular basal transcription machinery and histone acetyltransferases [8–12]. Interactions of BS69 with CR8 may affect the binding of EBNA2 with basal transcriptional machineries or histone acetyltransferases, thereby inhibiting the transactivation potential of EBNA2.

This study reveals that, whereas endogenous BS69 interacts with EBNA2 in EBV-infected B cells, the expression of BS69, at both mRNA and protein levels, is gradually down-regulated by EBNA2, which may be one of the strategies used by EBV to evade host defense. Given such a situation, it is impractical to determine the functional consequence of the BS69-EBNA2 interaction in cells through knockdown of endogenous BS69. Nevertheless, we show that ectopically expressed BS69_CC-MYND_ binds to EBNA2, leading to its enrichment at the EBNA2-targeted LMP1 promoter and inhibition of the EBNA2-mediated transcriptional activations. Through an inducible expression system, we further show that expression of BS69_CC-MYND_ inhibits the EBNA2-mediated proliferation of LCL cells. Therefore, this study suggests that restoration of BS69 expression in EBV-infected B cells may provide a mechanism to inhibit the proliferation of EBV-infected cells, which may have important implications in development of novel therapeutic strategies against EBV infection.

The coiled-coil domain of BS69 may have important biological functions other than EBNA2 binding. For instance, recent studies reveal that this region is responsible for mediating the interaction between BS69 and RNA splicing factor EFTUD2 [41], and that aggregation of BS69 is required for LMP1-mediated JNK signaling [42]. In addition to BS69, tandem arranged coiled-coil and MYND domains have been identified in AML1-ETO [43, 44] and DEAF-1 [45]. In AML1-ETO, oligomerization of the coiled-coil (also known as NHR2) domain is required for the interactions of AML1-ETO with ETO, MTGR1, MTG16 and E protein in the transcription factor complex (AETFC) [43, 44], and essential for AML1-ETO’s ability in inducing haematopoietic stem/progenitor cell self-renewal and leukaemogenesis [44]. In DEAF-1, the coiled-coil domain stabilizes the interaction between an unstructured region of DEAF1-1 and LMO4 protein [45]. In this context, this study adds a new example on how the coiled-coil domain of the MYND domain-containing proteins regulates their target recognition.

Genetic mutations within the BS69_MYND_ domain have been implicated in neuropsychiatric diseases [46, 47]. Our structural and biochemical studies therefore shed new light onto the pathological roles of the BS69 mutations in these diseases. For instance, the syndromic intellectual disability-associated mutations, Q547Δ and R560W [46, 47], are both located in the protein interaction sites of BS69_MYND_ (Fig 1D). Therefore, these two mutations may affect neural development through impairing the interaction between BS69 and its cellular partner(s). It would be interesting to identify the affected interacting partners in future studies.

## Materials and Methods

### Protein expression and purification

The DNA sequence encoding residues 440–562 (BS69_CC-MYND_) of mouse BS69/ZMYND11 was inserted into a modified PRSF-duet vector (Novagen), in which the BS69 fragments are separated from the preceding His6-SUMO tag by a ubiquitin-like protein (ULP1) cleavage site; and the DNA sequence encoding BS69 516–562 (BS69_MYND_) was inserted into a pGEX-6P-1 vector (GE Healthcare), preceded by a GST tag and a PreScission protease cleavage site. Note that the amino acid sequences of both fragments are identical to the corresponding regions of human BS69. The BS69_MYND_ mutants were constructed by site-directed mutagenesis and confirmed by DNA sequencing. The plasmid was then transformed into BL21 (DE3) RIL cell strain for overexpression. The bacterial cells were grown at 37°C and induced by 0.4 mM isopropyl β-d-1-thiogalactopyranoside (IPTG) when the cells density reached an optical density (OD600) of 0.6. After induction, the cells continued to grow at 20°C for overnight. The cells were then harvested and lysed, and the His6-SUMO- or GST-tagged BS69 fusion proteins were purified through a Ni-NTA column or a Glutathione Sepharose fast flow column (GE Healthcare), followed by removal of His6-SUMO tag and GST tag by ULP1 and PreScission protease cleavages, respectively. The tag-free BS69_MYND_ or BS69_CC-MYND_ was finally purified through size-exclusion (Superdex 200 16/60, GE Healthcare) chromatography in a buffer containing 20 mM Tris-HCl (pH 7.5), 50 mM Arginine hydrochloride, 50 mM Sodium Glutamate, 200 mM NaCl and 5 mM DTT. The protein samples used for crystallization were concentrated to ~10 mg/ml and stored at -80°C, and the samples for ITC analysis were concentrated to 2–4 mg/ml.

The EBNA2_381–389_ (NH2-SMPSLEPVL-CONH2) and EBNA2_435–445_ (NH2-EAPILFPDDWY-CONH2) peptides were synthesized from the proteomic facility of Tufts University. The EBNA2_381–445_ fragment was inserted into the PRSF-duet vector for expression, and sequentially purified through a Ni-NTA column, ULP1 proteolytic cleavage, ion exchange chromatography (HiTrap Q XL column, GE Healthcare) and size exclusion chromatography. The protein sample was concentrated to ~10 mg/ml in a buffer containing 20 mM Tris-HCl, 200 mM NaCl and 5 mM DTT for storage. The numbering systems used for EBNA2_381–389_, EBNA2_435–445_ and EBNA2_381–445_ peptides are based on full-length EBNA2 from EBV strain B95-8 [48]. For *in vivo* transcription assay, full-length or partial fragment of EBNA2 from EBV W91 strain [49] were used.

### Crystallization and structure determination

The BS69_CC-MYND_-EBNA2_381–389_ complex was prepared by direct mixing BS69_CC-MYND_ with the EBNA2_381–389_ peptide in a 1:2 molar ratio. Initial crystallization condition was identified using sparse-matrix screens (Hampton Research inc). The crystals were subsequently generated by hanging-drop vapor-diffusion method at 20°C, with the drops mixed from 0.5 μl of BS69_CC-MYND_-EBNA2_381–389_ solution and 0.5 μl of precipitant solution (19–23% PEGMME 350, 6% methanol, 0.1 M Tris-HCl, pH 8.0). The crystals were further improved by the microseeding method, and flash frozen with the cryoprotectant (17% PEGMME 350 and 20% glycerol) in liquid nitrogen before X-ray data collection.

Anomalous and native diffraction data sets for the BS69_CC-MYND_-EBNA2_381–389_ complex were collected on the BL 8.2.2 and 5.0.1 beamlines, respectively, at the Advanced Light Source (ALS), Lawrence Berkeley National Laboratory. The diffraction data were indexed, integrated and scaled using the HKL 2000 program. The structure of the BS69_CC-MYND_-EBNA2_381–389_ complex was solved using the AutoSol module embedded in the PHENIX software package [50]. The structural model was manually built using the program COOT [51] and was improved with iterations of manual model building and refinement using PHENIX. The final model was refined to 2.4 Å resolution using a native data set. The B-factors were refined with individual B values. The statistics for data collection and structural refinement for the BS69_CC-MYND_-EBNA2_381–389_ complex is summarized in S1 Table.

### ITC measurements

The EBNA2_381–389_ peptide with an additional Gly-Tyr dipeptide at the C-terminus (NH2-SMPSLEPVLGY-CONH2), the EBNA2_435–445_ peptide, and the EBNA2_381–445_ peptide were used for ITC assays. The BS69_MYND_ or BS69_CC-MYND_ domain (~0.1 mM each) and the peptides (~1 mM) were dialyzed against a buffer containing 20 mM Tris-HCl pH 7.5, 100 mM NaCl, 2 mM DTT. The peptide was titrated into the BS69_MYND_ or BS69_CC-MYND_ sample at 5°C using the microCal ITC200 instrument (GE healthcare). The ITC data was analyzed with the Origin 7.0 software using a one-site fitting model.

### Virus infection, quantitative real time PCR, and immunofluorescence microscopy

5x10^4^/per 100 μl primary B cells were cultured in RPMI 1640 supplemented with 15% fetal calf serum (FCS), 2 mM L-glutamine, and penicillin/streptomycin, and aliquoted into a 96-well plate. 100 μL previously purified EBV [52] or PBS (mock infection) was utilized as the inoculum. Cells were collected at 0, 1, 3, 5, and 7 days of post-infection (dpi) and BS69 mRNA expression in virus or mock -infected B cells were determined as the relative expression levels versus GAPDH by quantitative reverse transcription real time PCR (qRT-PCR). For Western blot analysis, a total of 5x10^5^ B cells with EBV infection were collected at 0, 1, 4, and 7 dpi, respectively. The primers for BS69 are GTCCACGGTATGCACCCTAAAGAG (F) and AACACCTCTCCAGGCAAATGG (R), whereas the primers for GAPDH internal control are AAGGTGAAGGTCGGAGTCAA (F) and AATGAAGGGGTCATTGATGG (R). The primers for chromatin immunoprecipitati on assays of the LMP1 promoter and GAPDH promoter control have been described previously [52]. The amount of ChIPed DNA was quantified by real time PCR and represented as % of input DNA. For Immunofluorescence analysis (IFA), cells were fixed in 2% paraformaldehyde (Sigma) and subjected to an immunostaining protocol using antibodies for BS69 (Santa Cruz), and EBNA2 (Santa Cruz). In co-immunostainings, Rhodamine-conjugated goat anti-mouse (for BS69) and FITC-conjugated donkey anti-goat (for EBNA2) (Kirkegaard & Perry Laboratories, Inc.) were used as fluorochromes, and DNA was counterstained with DAPI (Sigma).

### Co-immunoprecipitation, chromatin immunoprecipitation (ChIP), and western blot analyses

The transfection-mediated co-immunoprecipitation (Co-IP) assay was employed to identify the physical interaction of EBNA2 with BS69_CC-MYND_ using EBV-negative B lymphoma cells (BJAB). Cells were co-transfected with the expression plasmids of EBNA2 (E2) and flag- BS69_CC-MYND_ wild type, Q546A mutant (Q546A), or Q546A/W550A mutant (Q546A/W550A) and subjected to IP analysis using M2-conjugated sepharose (Sigma). The immunoprecipitated samples were subjected to SDS-PAGE, followed by Western blotting with antibodies for EBNA2 (Millipore) and M2 (Sigma), respectively. The antibody for actin internal control was purchased from Santa Cruz. The proteins were detected and visualized using an ECL detection kit (Millipore). ChIP assays were performed using BJAB cells that have been transfected with LMP1-promoter reporter plasmid (LMP1-Luc) and flag-BS69_CC-MYND_ wild type or Q546A, with or without E2 using M2-conjugated sepharose and IgG negative control (Millipore) following the previously described protocol [53). The protein levels of endogenous BS69 in the obtained cell lines were determined by Western blot using BS69 antibody (Santa Cruz biotechnology). The antibodies for immune blots are 6F9/60 (Novas Biologicals) for EBNA1, MABE8 (Millipore) for EBNA2, and C4 (Santa cruz) for actin internal control. The proteins were detected and visualized using an ECL detection kit (Millipore).

### Cell culture and reporter gene assays for EBNA2-mediated transcription activation

BJAB is an EBV negative B lymphoma cell line [26]. BJAB-E1 is an EBNA1 stably expressed BJAB cell line and BJAB-E2 is an EBNA2 stably expressed cell line. LCL#1–3 are three independent lymphoblastoid cell lines. EBV latently infected type I AKATA, AKATA (EBV+), and EBV-negative AKATA (EBV-) are two Burkitt’s Lymphoma (BL) cell lines [26, 32]. All the above cell lines were cultured in RPMI-1640 (Life Technology) supplemented with 10% fetal calf serum (FCS) (Life Technology). HeLa cells were maintained in Dulbecco's Modified Eagle Medium supplemented with 10% fetal bovine serum at 37°C under 5%CO2.

The BS69_CC-MYND_ dual domain, wild-type or the Q546A mutant, were inserted into a pCBS/FLAG mammalian expression vector [54], in which the BS69 fragments are preceded by a FLAG-tag. In the case of the Q546A/W550A BS69_CC-MYND_ expression plasmid, the flanking DNA fragment was subcloned into the pSG5-Flag vector [55]. The EBNA2 expression vector harboring PXLXP1 or PXLXP2 mutations was generated by carrying out the PCR-based mutagenesis protocol provided by the manufactory (Stratagene). In each case, the codons of two prolines were replaced with the codons of alanine simultaneously. For EBNA2-dependent transcription reporter assays, 10 μg of the expression vector of EBNA2 (E2), 5 μg flag-BS69_CC- MYND_ wild type, or Q546A plasmid, and 5 μg LMP1-Luc or Cp-Luc reporter plasmid, and 1 μg CMV-βGal control plasmid were co-transfected into BJAB cells following the protocol as described previously [52]. For the parallel control, 10 μg EBNA1 expression plasmid and 5 μg OriP-Luc reporter plasmid were used instead [53]. Luciferase and β-Gal activities were assayed by Orion L (Berthold). A DNA fragment encoding residues 375–465 of EBNA2 from EBV W91 strain [49] containing CR7 and CR8 of EBNA2 were inserted into pCD-Gal4(1–147) vector to generate pCD-Gal4-EBNA2(375–465), which encodes a GAL4-EBNA2 fusion protein. Hela cells in 6-well plates were transfected using Lipofectamine 2000 (Life Technologies) with 500 ng pG5-TK-Luc (a gift from Dr. Yang Shi, Harvard Medical School), 200 ng of pCMV-β-Galactosidase, 500 ng pCD-Gal4-EBNA2(375–465), and the indicated amounts of pCbS/Flag-BS69_CC-MYND_ wild-type or Q546A mutant. In control transfection assays 500ng of the pCDNA 3.1(+) empty vector, or the derivatives pCD-Gal4(1–147) vector expressing the Gal4 DNA-binding domain (DBD), or the pCD-Gal4-VP16 vector expressing the Gal4-VP16 fusion activator, were used instead of pCD-Gal4-EBNA2, as indicated. Cell extracts were prepared after 48h and luciferase, beta-galactosidase assays, and Western blotting were performed essentially as previously described [54]. Luciferase activities were normalized to beta-galactosidase activities and results are the means ± S.D. of a minimum of 3 independent experiments each in triplicates.

### Retrovirus vector-mediated gene inducible system and cell viability assays

The flanking fragments of flag-BS69_CC-MYND_ wild type, Q546A, and Q546A/W550A were subcloned inframe into the downstream of the destabilizing domain (DD) in the context of the modified pQCXIP expression vector (Clontech), respectively. Retrovirus vectors harboring DD-flag BS69_CC-MYND_ wild type, Q546A, or Q546A/W550A, DD-GFP, or GFP alone were produced and harvested by performing the protocol provided by the manufacture. 1 mL of virus suspension was used to transduce 1 mL LCL (10^6^ per mL) or control BJAB (10^6^ per mL) cells following a cell culture procedure with selection of 5 ng/mL puromycin. In addition, the culture medium was supplemented with 8 μg/mL polybrene (Sigma) in order to enhance virus transduction efficiency. For the cell viability assays, 10^5^ LCL or BJAB cells per mL were aliquoted into 6-well plate. The DD-fusion was induced by treatment of 0.1 mM Shield 1 and viable cells were counted by cellometer K2 (Nexcelom) using the trypan blue exclusion method every 24 hours (hrs) for five consecutive days.

## Supporting Information

S1 Fig ITC analysis of the interaction between BS69 and EBNA2. (A) Summary of the ITC parameters for the interactions between BS69 domains and EBNA2 peptides. ^a^The average and standard deviations of these values were derived from three independent measurements. Otherwise, the parameters were determined from single measurements, with the uncertainties estimated from curve fitting. ^b^The *K*_d_ value was estimated by fixing the parameter of the stoichiometric ratio (N) to 1. N.M.: not measurable. (B-E) ITC analysis of the binding of BS69_MYND_ and EBNA2_435–445_ (B), BS69_MYND_ Q546A and EBNA2_435–445_ (C), BS69_CC-MYND_ Q546A and EBNA2_435–445_ (D), and BS69_CC-MYND_ Q546A/W550A and EBNA2_435–445_ (E). In (D-E) the ITC parameters could not be reliably determined. However, the heat measurements are significantly lower than those of Fig 2H.
(TIF)

S2 Fig Model of the BS69_CC-MYND_ homodimer binding to the two PXLXP motifs (green) of EBNA2 simultaneously. The corresponding domains of BS69 and EBNA2 are labeled. This model is consistent with our structural observation that each BS69 MYND domain binds to one PXLXP motif of EBNA2. On the other hand, it does not consider the effect of EBNA2 dimerization, which conceivably will further oligomerize the BS69-EBNA2 complex.
(TIF)

S3 Fig *In vivo* transcription assays of G5-TK-Luc reporter gene. (A) The expression of Flag-tagged wild-type or Q546A BS69_CC-MYND_ in HeLa cells was analyzed by Western blotting using anti-flag M2 monoclonal antibody. The expression of β-actin was used as control. (B) Relative transcription activation by GAL4 DBD, Gal4-EBNA2 and Gal4-VP16 was analyzed in transfected HeLa cells in the absence or presence of 10 ng expression vector for wild-type BS69_CC-MYND_ or its Q546A mutant. Fold activation is relative to reporter gene activity in the absence of Gal4 activator (set to 1). (C) The relative transcription of the G5-TK-Luc reporter in Gal4-EBNA2 transfected Hela cells is shown as a function of the amount of BS69_CC-MYND_ (wild-type or Q546A mutant) vector used.
(TIF)

S4 Fig PXLXP2 is critical for BS69 mediated down-regulation of EBNA2 dependent transcription. (A) The expression plasmid of wild-type, PXLXP1 mutant or PXLXP2 mutant EBNA2 was cotransfected with LMP1-Luc and internal control CMV-β gal. The EBNA2 induced activity of the LMP1-Luc reporter plasmid was shown. (B-D) The expression plasmid of EBNA2 (B), EBNA2 PXLXP1 mutant (C), or EBNA2 PXLXP2 mutant (D) was cotransfected with Flag-BS69cc_-MYND_ WT, Q546A, or Q546A/W550A and LMP1-Luc and internal control CMV-β gal. The resulting activity produced by each transfectant relatively to the intrinsic activity of EBNA2, PXLXP1 mutant, or PXLXP2 mutant was shown. The expression levels for all the indicated transfected plasmids and actin were shown.
(TIF)

S1 Table Crystallographic statistics for the BS69_CC-MYND_-EBNA2_381–389_ complex. (DOCX)
