# Supplementary figures and images for: BS69/ZMYND11 C-Terminal Domains Bind and Inhibit EBNA2
Source: PLoS Pathog. 2016 Feb 4;12(2):e1005414. doi: 10.1371/journal.ppat.1005414 (PMC4742278; doi:10.1371/journal.ppat.1005414)

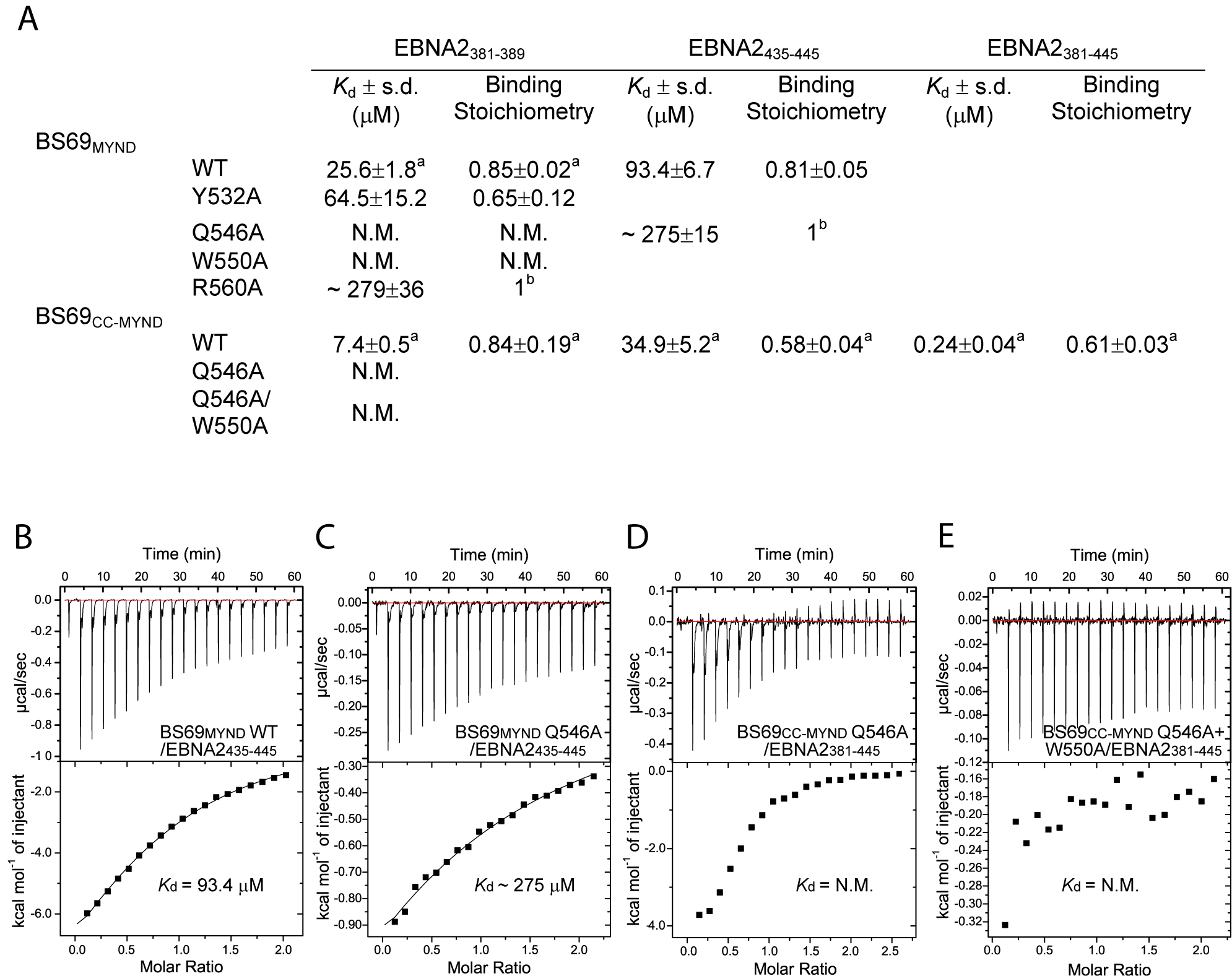

Supplement: S1 Fig — (A) Summary of the ITC parameters for the interactions between BS69 domains and EBNA2 peptides. aThe average and standard deviations of these values were derived from three independent measurements. Otherwise, the parameters were determined from single measurements, with the uncertainties estimated from curve fitting. bThe K d value was estimated by fixing the parameter of the stoichiometric ratio (N) to 1. N.M.: not measurable. (B-E) ITC analysis of the binding of BS69MYND and EBNA2435–445 (B), BS69MYND Q546A and EBNA2435–445 (C), BS69CC-MYND Q546A and EBNA2435–445 (D), and BS69CC-MYND Q546A/W550A and EBNA2435–445 (E). In (D-E) the ITC parameters could not be reliably determined. However, the heat measurements are significantly lower than those of Fig 2H. (TIF) [file ppat.1005414.s001.tif]

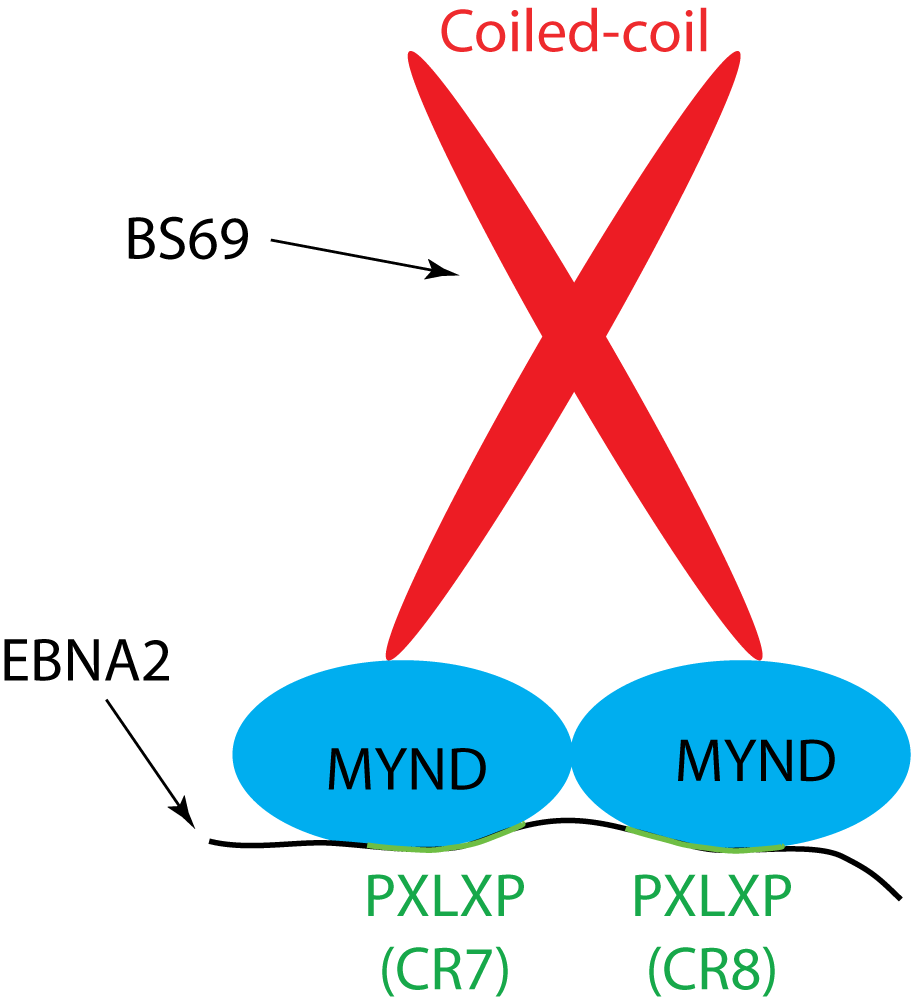

Supplement: S2 Fig — The corresponding domains of BS69 and EBNA2 are labeled. This model is consistent with our structural observation that each BS69 MYND domain binds to one PXLXP motif of EBNA2. On the other hand, it does not consider the effect of EBNA2 dimerization, which conceivably will further oligomerize the BS69-EBNA2 complex. (TIF) [file ppat.1005414.s002.tif]

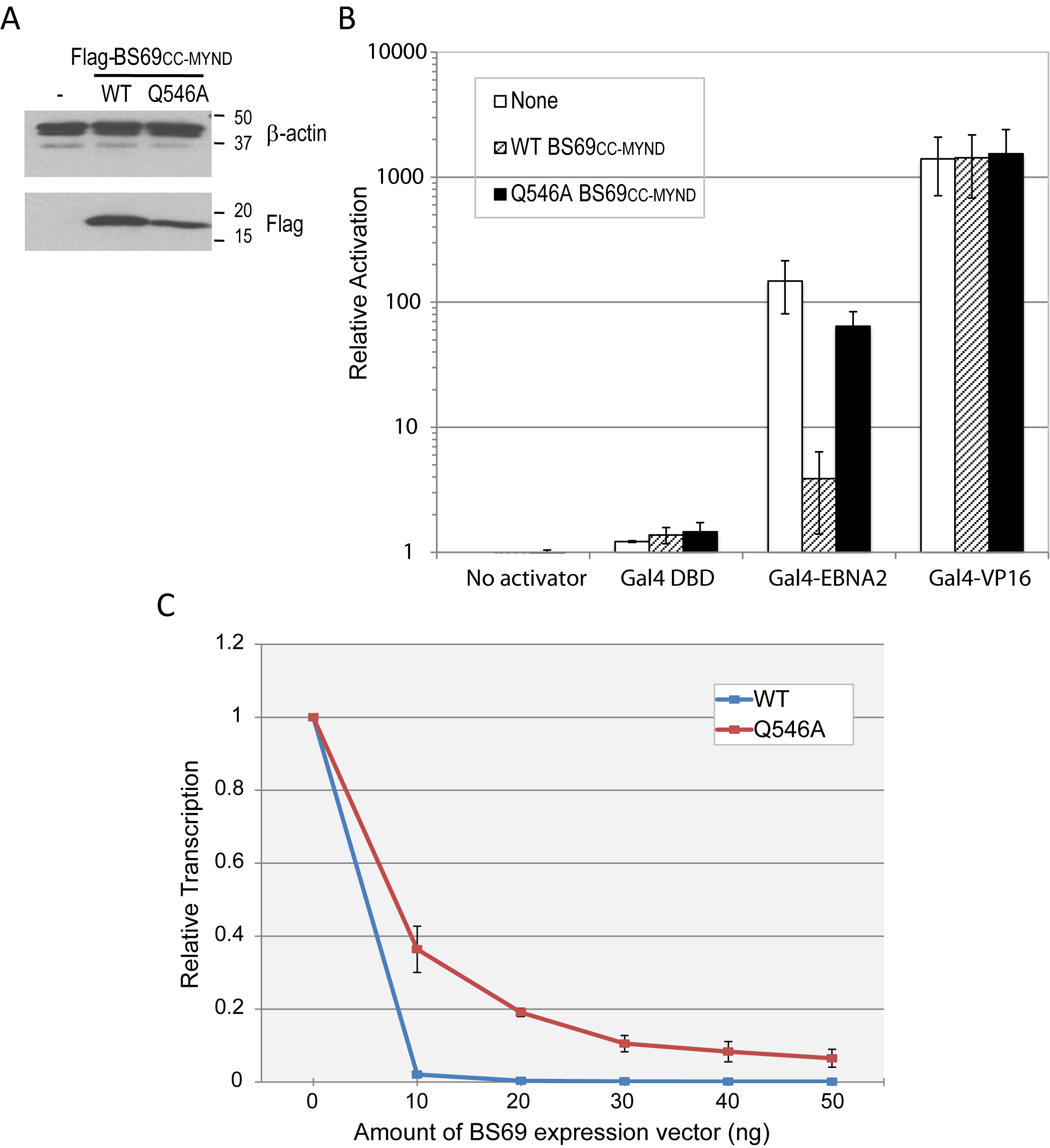

Supplement: S3 Fig — (A) The expression of Flag-tagged wild-type or Q546A BS69CC-MYND in HeLa cells was analyzed by Western blotting using anti-flag M2 monoclonal antibody. The expression of β-actin was used as control. (B) Relative transcription activation by GAL4 DBD, Gal4-EBNA2 and Gal4-VP16 was analyzed in transfected HeLa cells in the absence or presence of 10 ng expression vector for wild-type BS69CC-MYND or its Q546A mutant. Fold activation is relative to reporter gene activity in the absence of Gal4 activator (set to 1). (C) The relative transcription of the G5-TK-Luc reporter in Gal4-EBNA2 transfected Hela cells is shown as a function of the amount of BS69CC-MYND (wild-type or Q546A mutant) vector used. (TIF) [file ppat.1005414.s003.tif]

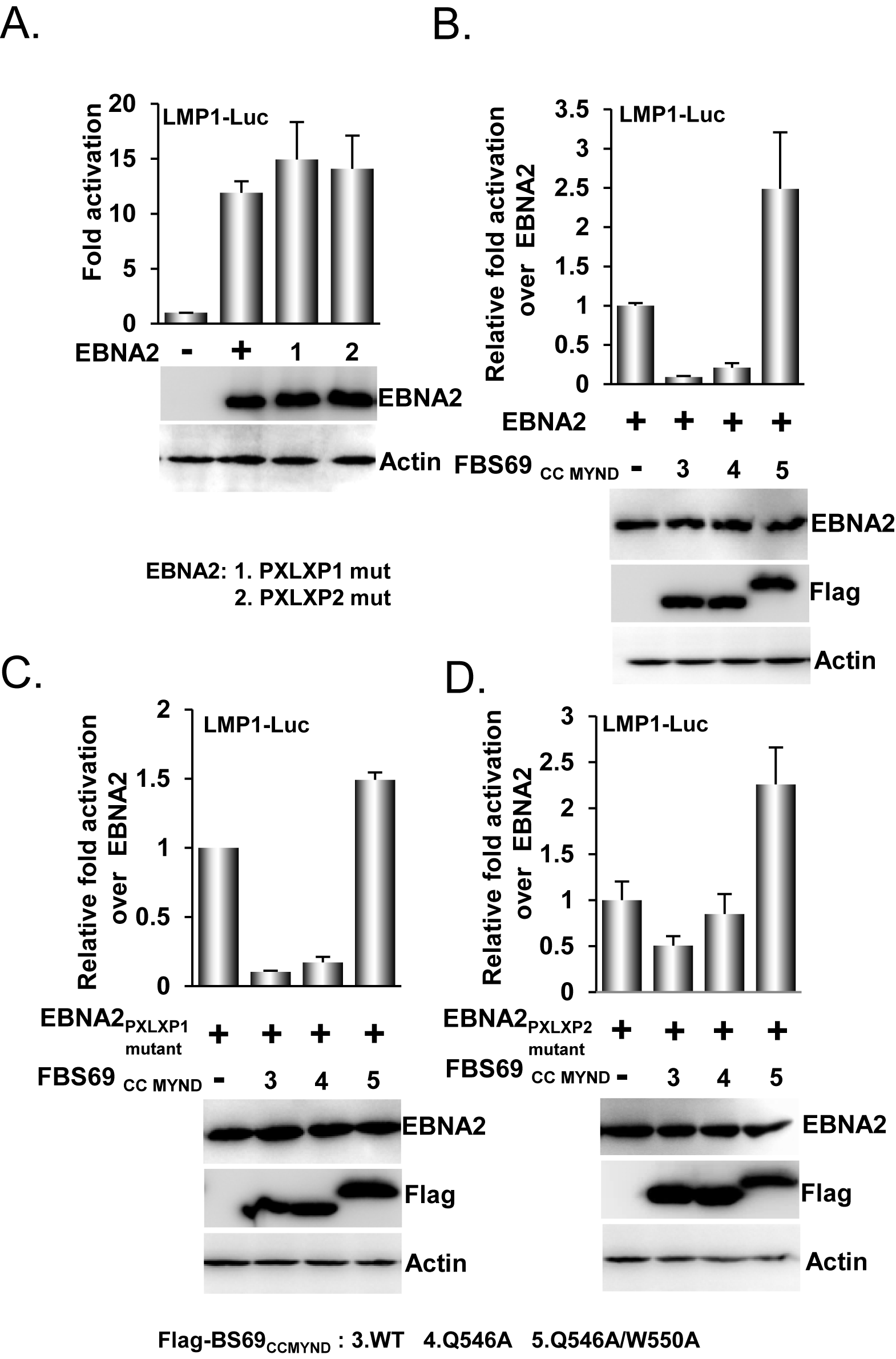

Supplement: S4 Fig — (A) The expression plasmid of wild-type, PXLXP1 mutant or PXLXP2 mutant EBNA2 was cotransfected with LMP1-Luc and internal control CMV-β gal. The EBNA2 induced activity of the LMP1-Luc reporter plasmid was shown. (B-D) The expression plasmid of EBNA2 (B), EBNA2 PXLXP1 mutant (C), or EBNA2 PXLXP2 mutant (D) was cotransfected with Flag-BS69cc-MYND WT, Q546A, or Q546A/W550A and LMP1-Luc and internal control CMV-β gal. The resulting activity produced by each transfectant relatively to the intrinsic activity of EBNA2, PXLXP1 mutant, or PXLXP2 mutant was shown. The expression levels for all the indicated transfected plasmids and actin were shown. (TIF) [file ppat.1005414.s004.tif]
