# Supplementary material for: BS69/ZMYND11 C-Terminal Domains Bind and Inhibit EBNA2
Source: PLoS Pathog. 2016 Feb 4;12(2):e1005414. doi: 10.1371/journal.ppat.1005414 (PMC4742278; doi:10.1371/journal.ppat.1005414)
Supplement: S1 Table — (DOCX) [file ppat.1005414.s005.docx]

|  | BS69_CC-MYND_-EBNA2_381-389_ | |
| --- | --- | --- |
| **Data collection** |  |  |
|  | Crystal 1 | Crystal 2 |
| Wavelength (Å) | 0.9774 | 1.2818 |
| Space group | *P2_1_* | *P2_1_* |
| Cell dimensions |  |  |
| *a*, *b*, *c* (Å) | 58.5, 38.2, 82.7 | 58.5, 38.0, 82.2 |
| * α* *β* *γ* (°) | 90, 104.4, 90 | 90, 97.9, 90 |
| Resolution (Å)* | 50-2.4 (2.49-2.4) | 50-2.6 (2.69-2.6) |
| *R*_sym_ or *R*_merge_* | 9.6 (36.2) | 15.2 (41.9) |
| *I*/σ*I* | 12.4 (2.3) | 8.6 (2.2) |
| Completeness (%)* | 99.4 (98.8) | 97.0 (95.2) |
| Redundancy* | 3.2 (2.9) | 2.9 (2.4) |
| Unique reflections* | 14,546 (1,408) | 10931 (1075) |
|  |  |  |
|  |  |  |
| **Refinement** |  |  |
| Resolution (Å) | 44.6-2.4 |  |
| No. reflections | 14,519 |  |
| *R*_work_/*R*_free_ | 22.2/26.2 |  |
| No. atoms |  |  |
| Protein | 1946 |  |
| Peptide | 124 |  |
| Zinc | 4 |  |
| Water | 107 |  |
| B-factors |  |  |
| Protein | 48.4 |  |
| Peptide | 46.1 |  |
| Zinc | 31.8 |  |
| Water | 43.5 |  |
| R.m.s deviations |  |  |
| Bond lengths (Å) | 0.005 |  |
| Bond angles (º) | 0.874 |  |
| Ramachandran plot |  |  |
| Favoured regions (%) | 96 |  |
| Allow regions (%) | 4 |  |
| Outliers (%) | 0 |  |

*Highest resolution shell is shown in parenthesis.
